# A Rare Complication Reversed: Successful Testicular Revascularization After Inguinal Hernia Repair

**DOI:** 10.7759/cureus.105743

**Published:** 2026-03-23

**Authors:** Cansu Yalçın, Kemal Yüksel, Sebat Karamürsel

**Affiliations:** 1 Department of Plastic and Reconstructive Surgery, Ordu State Hospital, Ordu, TUR; 2 Department of Plastic and Reconstructive Surgery, Etlik City Hospital, Ankara, TUR

**Keywords:** inguinal hernia, microvascular anastomosis, revascularization, testicle, testicular atrophy

## Abstract

Testicular atrophy is a recognized complication that may occur following inguinal hernioplasty, typically resulting from injury or the avulsion of the testicular vessels. The ligation of the testicular vessels during inguinal hernioplasty is exceedingly rare and is generally encountered in complicated cases.

In the present case report, we describe a patient who underwent immediate testicular revascularization following the ligation of the testicular vessels during a complex and adherent inguinal hernioplasty. After the successful microvascular anastomosis of the testicular vessels, no evidence of testicular atrophy was observed during follow-up. Doppler ultrasonography (USG) demonstrated preserved and normal testicular perfusion. Interestingly, the Doppler USG of the contralateral testis revealed minimal atrophy, which was most likely due to a preexisting condition.

This case highlights the critical importance of the prompt recognition and repair of testicular arterial injury, particularly in young patients, to prevent irreversible ischemic damage and preserve gonadal function.

## Introduction

Intraoperative damage to the testicular vessels, although rare, represents a serious event. This is considered to be the result of an acute thrombosis of the pampiniform venous plexus rather than an acute arterial injury, given the collateral blood supply to the testis through the inferior epigastric, vesical, prostatic, and scrotal arteries [1]. Indeed, some reports indicate that testicular ischemia does not develop in up to one-third of cases where the spermatic cord has been intentionally ligated [2]. Nevertheless, the risk of ischemic orchitis or testicular atrophy remains significant, particularly in young patients, where the physiological and reproductive implications are more pronounced. Consequently, in the management of younger patients, prioritizing the surgical reconstruction of the testicular vasculature is often a more prudent clinical approach. In clinical practice, these vascular injuries typically occur during the difficult dissection of large hernia sacs that are densely adherent to the spermatic cord. Performing an immediate revascularization is clinically vital not only to prevent irreversible testicular atrophy but also to protect the patient's fertility and endocrine function.

This report outlines the surgical restoration of blood supply to the testis in a 44-year-old individual, after an unintentional vascular injury occurred during a primary inguinal hernia repair. The case underscores the importance of meticulous surgical technique, the early identification of vascular compromise, and the feasibility of microvascular repair in preserving testicular viability in young male patients. While previous reports of intraoperative transection involved delayed revascularization, our case demonstrates the critical value of an immediate, on-site microvascular repair to minimize ischemia time and ensure complete testicular survival.

## Case presentation

A 44-year-old male patient presented to the emergency department with complaints of swelling and pain in the right groin and scrotum. After a general surgery consultation, the patient was hospitalized for an open elective inguinal hernia operation. On the following day, the patient underwent an open surgical exploration. Intraoperatively, an indirect inguinal hernia was confirmed. Dense adhesions were observed between cord structures and the hernia sac. During the careful dissection of the hernial sac from cord structures at the level of the deep inguinal ring, the testicular artery and vein were inadvertently ligated. The plastic surgery team was immediately consulted and joined the operating table within just 15 minutes of the vascular injury. Upon initial assessment, the testis exhibited no macroscopic signs of ischemia, such as discoloration or pallor, owing to this remarkably prompt intervention.

**Figure 1:**
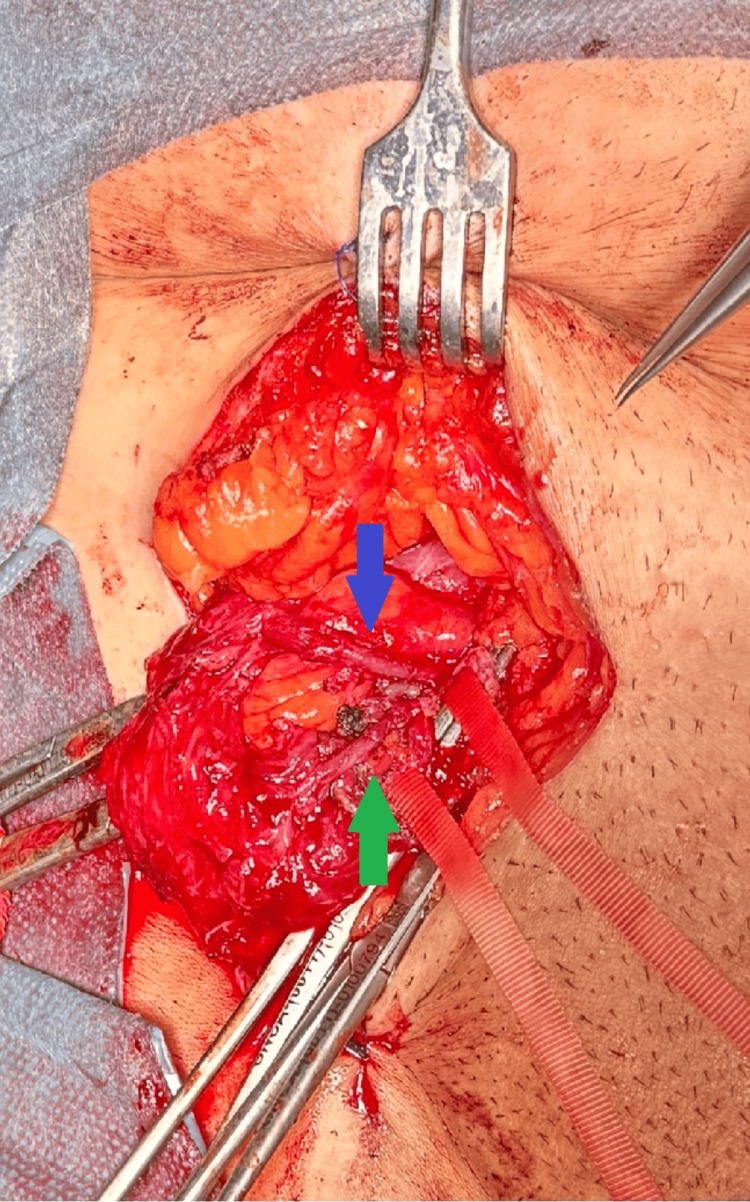
Distant view of the surgical field Distant view of the surgical field showing the anastomosed vessels, with the reconstructed arterial and venous segments clearly visualized within the operative area. Arterial anastomosis is highlighted by the blue arrow, whereas the venous anastomosis is identified by the green arrow

**Figure 2:**
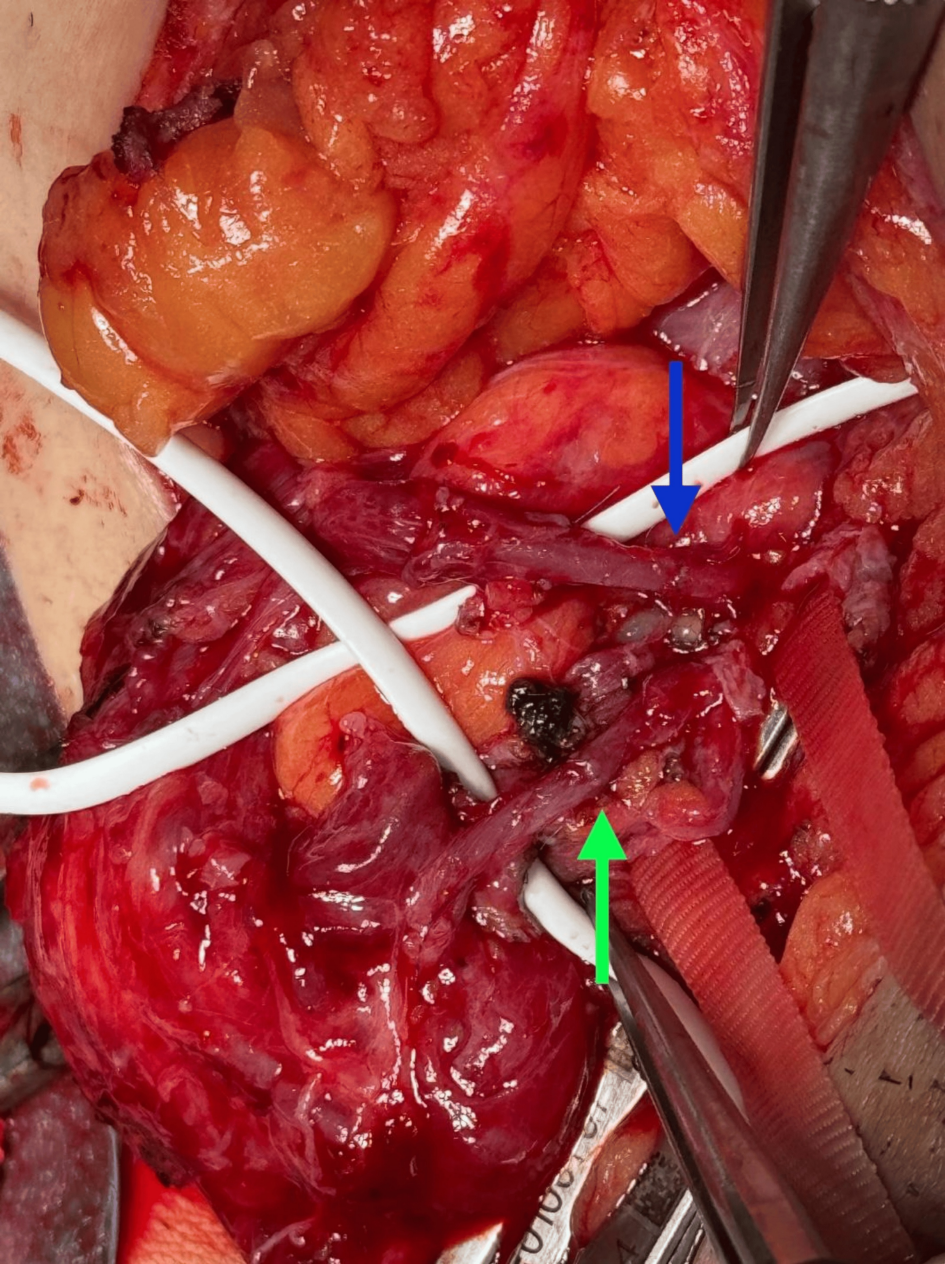
Magnified view of the anastomoses Arterial anastomosis is highlighted by the blue arrow, whereas the venous anastomosis is identified by the green arrow. Both anastomoses were performed using a 10-0 Prolene

The vas deferens was intact. The testicular artery and vein were identified, and the coagulated segments were excised under the operating microscope. The vessels were prepared for anastomosis, and then, microvascular anastomosis was performed with 10/0 Prolene sutures (Ethicon Inc., Raritan, NJ) (Figure 1). Surgical exploration and arterial anastomosis were successfully completed within one hour of the initial injury, while the venous anastomosis was performed during the subsequent hour. The total revascularization of the testicle was thus achieved approximately two hours after the division of the blood vessels (Figure 2). The milking test confirmed the patency of the vessels, and the case was handed back to the general surgery team. The hernia repair was completed without the use of a mesh due to the vascular anastomosis.

The patient received intravenous dextran for five days and subcutaneous enoxaparin during hospitalization. The patient's recovery progressed without incident, allowing for hospital discharge in stable condition on the seventh postoperative day.

**Figure 3:**
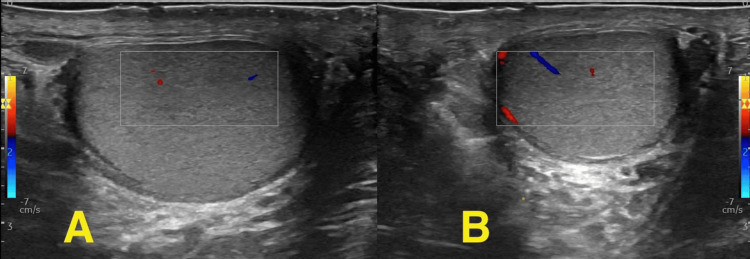
Doppler ultrasound Doppler ultrasound performed at the eighth postoperative month demonstrates normal blood flow in the right testis (A, right testis; B, left testis)

**Figure 4:**
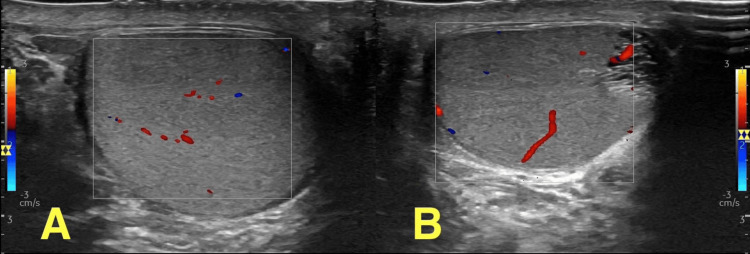
Doppler ultrasound Doppler ultrasound shows preserved blood flow in the left (unaffected) testis, despite the presence of testicular atrophy, which is presumed to have been preexisting. These findings highlight the importance of testicular vessel repair in young patients due to the potential risks involved (A, right testis; B, left testis)

In the postoperative eighth month, the testis appeared normal in size, consistency, and sensation on clinical examination. A follow-up Doppler ultrasonography (USG) confirmed normal vascular flow in the right testicle, with no evidence of ischemia, inflammation, or atrophy (Figure 3). However, a Doppler ultrasonography of the contralateral testis revealed minimal atrophy, which was interpreted as a preexisting condition (Figure 4).

## Discussion

Overzealous dissection during hernia surgery and the dislocation of the testis can increase the risk of testicular atrophy, and the thrombosis of the spermatic cord veins from surgical dissection trauma can lead to testicular atrophy [3]. However, the arterial interruption alone may not always lead to ischemia because of these collaterals. Orchitis is believed to occur more frequently after open procedures, particularly in large indirect or recurrent inguinal hernias, due to the greater manipulation of the spermatic cord beyond the pubic tubercle and during the dissection of the distal hernia sac [1]. A 1% incidence of testicular atrophy in 28,760 open inguinal hernia repairs was reported [4], while it is noted that testicular problems, including pain, swelling, and orchitis, occur in 0.9%-1.5% of laparoscopic inguinal hernia repairs [5]. However, the use of cautery in close proximity to the cord structures during a laparoscopic approach may increase the risk of venous thrombosis [6].

In addition to vascular compromise caused by extensive dissection, testicular vessel injury may also occur secondary to the transection of the spermatic cord, as in our presented case. The interruption of spermatic vasculature yields a triad of potential sequelae: a gangrenous testis, ischemic orchitis and slow testicular atrophy, or an entirely asymptomatic presentation with no structural damage [7]. This type of injury is distinct from the usual venous congestion or thrombosis seen after standard hernioplasty and represents a complete interruption of both arterial inflow and venous outflow to the testis. If not promptly recognized and managed, such injuries can rapidly lead to testicular ischemia and infarction. Interestingly, existing literature indicates that nearly a third of testes maintain their viability without exhibiting ischemic alterations, even following the intentional ligation of the spermatic cord [2].

There is an ongoing lack of consensus regarding whether routine orchiectomy is the optimal management for spermatic cord trauma, a debate further compounded by insufficient data detailing the precise incidence and temporal onset of subsequent necrotic orchitis [8]. In experimental rat models, restricting testicular perfusion for as little as 15 minutes yields permanent ischemic damage at two months, effectively halving both germinal epithelial volume and overall spermiogenic capacity [9]. Therefore, early identification and immediate microvascular repair are essential to preserve testicular viability, especially in young patients with high reproductive potential. In the only reported case in the literature of a spermatic cord transection during inguinal hernia repair, revascularization was performed six hours later, and no testicular atrophy was observed [7]. In our case, arterial anastomosis was completed within one hour of the testicular vessel injury, and venous anastomosis was performed during the following hour. Doppler ultrasonography subsequently demonstrated adequate testicular perfusion, with no evidence of testicular atrophy. Interestingly, a Doppler USG of the contralateral testis revealed minimal atrophy, which was most likely due to a preexisting condition. In the younger male demographic, encountering such vascular injuries warrants a definitive attempt at testicular salvage, making it the preferable alternative to organ removal [10].

## Conclusions

The early recognition and immediate microsurgical repair of the testicular vessels are crucial to restore testicular perfusion and prevent ischemic atrophy. This case demonstrates that timely revascularization can result in preserved testicular viability and function. Given the potential for such complications, vascular repair should be considered indicated in young patients to maintain gonadal integrity and fertility potential.
